# Association of Psychological Stress With Skin Symptoms Among the Population in Saudi Arabia: A Cross-Sectional Study

**DOI:** 10.7759/cureus.48657

**Published:** 2023-11-11

**Authors:** Elaf R Altalhi, Saja A Felimban, Wesam S Alharbi, Warif M Albogami, Ward M Malibari, Sarah S Alharbi, Yosra Z Alhindi

**Affiliations:** 1 Department of Medicine and Surgery, College of Medicine, Umm Al-Qura University, Makkah, SAU; 2 Department of Pharmacy, Umm Al-Qura University, Makkah, SAU; 3 Department of Pharmacology and Toxicology, Faculty of Medicine, Umm Al-Qura University, Makkah, SAU

**Keywords:** anxiety, ksa, survey, skin symptoms, psychological stress

## Abstract

Background and aim

Studies have shown a significant relationship between psychological stress (PS) and health, and it is widely believed that factors such as stress and anxiety may not only be the result of certain diseases but can also cause and exacerbate some diseases. There is a lack of research on PS and its association with other skin conditions. Thus, this study aimed to examine the association of PS with skin symptoms using objective scales in the general population in the Kingdom of Saudi Arabia (KSA).

Methods

A cross-sectional study was carried out between February 6, 2023, and April 4, 2023. We administered an electronic questionnaire survey, consisting of Cohen’s perceived stress scale and a self-reported skin complaint questionnaire, distributed via an online platform in the KSA. In all, 629 completed questionnaires were returned. Statistical analysis was conducted using RStudio. The results were presented as beta coefficients and their respective 95% confidence intervals (95% CIs). A p-value of <0.05 indicated statistical significance.

Results

The majority of the participants were female (71.7%, n=388), of Saudi nationality (93.2%, n=504), had a bachelor’s degree (68.6%, n=371), were aged 18-29 years (58.8%, n=318), and were residing in the Western region (39.9%, n=216). Acne (26.8%, n=145) and eczema (12.8%, n=69) were the most commonly reported skin conditions. The stress level was low in 30.5% of the respondents (n=165), moderate in 64.9% of the respondents (n=351), and high in 4.6% of the respondents (n=25). The average skin symptom score was significantly predicted by the presence of psoriasis (p < 0.001), eczema (p < 0.001), acne (p < 0.001), rash (p < 0.001), and baldness (p = 0.041). Furthermore, the average skin symptoms scores were significantly higher among participants with high stress (median = 1.6, interquartile range (IQR) = 1.4, 1.8) than among those with moderate (median = 1.4, IQR = 1.2, 1.8) and low stress (median = 1.4, IQR = 1.1, 1.4, p < 0.001).

Conclusion

The general population of Saudi Arabia reported multiple skin symptoms associated with stress. PS can cause various common skin conditions including loss of hair, eczema, and acne. This study highlights the importance of assessing common skin problems in the general population in the KSA and their strong association with PS. Various skin conditions including loss of hair, eczema, and acne can be caused by PS. Dermatologists should be aware of the context of PS when assessing patients with these conditions.

## Introduction

Numerous studies have shown a significant relationship between psychological stress (PS) and health. PS has been linked to conditions including, but not limited to, irritable bowel syndrome, bronchial asthma, and cardiovascular disorders [1-3]. PS is the result of the body's inability to appropriately respond to actual or imagined physical, mental, or emotional demands [4]. PS occurs when an individual perceives that the environmental demands exceed their adaptive capacity [5]. Upon perception of PS, two major neuroendocrine systems, the sympathetic nervous system and the hypothalamic-pituitary-adrenal (HPA) axis, are activated as part of the central stress response to PS to enable the organism to adapt to it [6]. This stress response leads to various consequences for the body's physiological system. Chronic stress exposure is typically thought to be associated with a greater risk of long-term health issues than acute stress because permanent and/or prolonged physiological, emotional, and behavioral responses are most likely to be elicited by chronic stress, which may play a role in the etiology and or exacerbation of diseases [7].

PS has several negative effects on the skin, such as impaired stratum corneum cohesion, disrupted permeability barrier function, delayed wound healing, altered antimicrobial properties of the epidermal barrier, compromised epidermal innate immunity, and impaired cutaneous homeostasis [8-12]. Consequently, these changes affect skin immunity, encourage the progression of infections, and lead to chronic inflammatory skin conditions. Additionally, psychosocial factors may contribute to the etiology or exacerbation of specific dermatological conditions such as alopecia areata, psoriasis, acne, atopic dermatitis, and urticaria [13-17].

However, even though many studies on this topic have been published, there is a lack of controlled studies using established methods to measure stress [18]. In addition, the prevalence of psychological distress is difficult to determine using previous studies because of the variations in the scales used to assess distress, differences in the time periods of symptom reporting, and variations in the cutoff points used to dichotomize distress scores and identify persons with pathological distress. In the general population, the prevalence of PS is reported to range between 5% and 27% [19].

Some studies assessed the relationship between PS and acne and hair loss in the KSA, but there is a lack of research on the association of PS with other skin conditions. Thus, this study aimed to determine the association between PS and skin symptoms in the general population in the KSA.

## Materials and methods

Study design and sample population

This was a web-based descriptive cross-sectional study that lasted from February 6, 2023 to April 4, 2023. The minimum sample size required for this study was calculated by OpenEpi version 3.0118, in consideration of the following: the population of Saudi Arabia is about 35,34 million inhabitants [20]. Considering a confidence interval (CI) of 95%, anticipated frequency of 50%, and design effect of 1, a sample size of 385 participants was obtained. To account for possible data loss, a sample of 629 participants was required. All the residents of the KSA who were eligible to answer our questionnaire were included in the study by Google form required to answer tool. However, we excluded members who were not eligible, refused to participate, or had any mental disorders.

Questionnaire structure

The questionnaire was designed using Google Forms and distributed electronically via different social media platforms. The questionnaire was translated to Arabic (the native language of the participants) before dissemination, and the responses were translated back to English for analysis and publication. The questionnaire was inspired by a previous study conducted at the College of Medicine, King Saud University, Riyadh, KSA [21], with some modifications made to the section on demographic data.

The questionnaire was divided into three main sections. The first section consisted of questions on sociodemographic information, with nine questions on gender, nationality, age, residence area, marital status, educational level, occupation, monthly income, and history of skin conditions.

The second section consisted of the self-reported skin complaints questionnaire (SSCQ), which is a validated 10-item survey, used to identify self-reported skin symptoms and predict clinical skin morbidity. It has been used in several large cross-sectional community-based studies [22-25]. It includes a multiple-choice grid table with 10 items, scored using a four-point scale: "1" denotes (No), "2" denotes (Yes, a little), "3" denotes (Yes, quite a lot), and "4" denotes (Yes, very much) [22]. We modified the SSCQ by adding the following six items under the 10th item “other skin problems”: hair shedding, hair pulling, nail-biting, itchy scalp, scaly scalp, and facial scales. The average skin symptom score (mean, standard deviation) for each patient was obtained by summing the scores on the 15 items.

The third section consisted of Cohen’s perceived stress scale (PSS) questionnaire. A modified version of the 10-item PSS questionnaire was distributed in Arabic and English [7,26]. The PSS includes questions on stressful situations in a person’s life over the past month. Each question is scored on a five-point scale: "0" for (never), "1" for (almost never), "2" for (sometimes), "3" for (fairly often), and "4" for (very often). Reverse coding was performed for 4 questions: (items 4, 5, 7, and 8). The overall PSS score ranges from 0 to 40. A numerical variable was generated by summing the scores on the 10 items. This numerical variable was converted into a categorical variable with three categories: low stress (0-13 points), moderate stress (14­-26 points), and high stress (27-40 points). These categories were coded as: "0" for low stress, "1" for moderate stress, and "2" for high stress [7,26].

Statistical analysis

Statistical analysis was conducted using RStudio (R version 4.2.2). Categorical variables were presented as frequencies and percentages, and continuous data were expressed as medians and interquartile ranges (IQRs). The demographic data and categorical variables of the stress level were subjected to cross-tabulation, and the statistical significance was tested using a Fisher's Exact Test for count data with simulated p-value based on 2000 replicates. The Mann-Whitney test was used to compare the average skin symptoms across demographic variables with two categories (gender and nationality), while the Kruskal-Wallis test was used to compare the variables with three or more categories (age, residential area, marital status, educational level, occupation, and monthly income). Independent predictors of high skin symptoms score were assessed by incorporating the significantly associated variables from the inferential analysis into a multivariable general linear model. The results were presented as beta coefficients and their respective 95% CIs. A p-value of <0.05 indicated statistical significance.

Ethical approval and informed consent

Ethical approval was obtained from the Biomedical Ethics Committee of the Faculty of Medicine at UQU, Makkah, KSA (approval number HAPO-02-K-012-2023-02-1437); the study was conducted in accordance with the Declaration of Helsinki. Electronic informed consent was obtained from each participant prior to administering the questionnaire. Confidentiality was ensured. The names or phone numbers of the participants were not collected.

## Results

Demographic characteristics and the history of skin conditions

Initially, we collected 629 responses on the online platform. However, we excluded 14 records of those who disagreed to participate and 74 responses for participants with mental disorders. Eventually, a total of 541 responses were analyzed. The majority of participants were females (71.7%, n=388), Saudis (93.2%, n=504), and had obtained a bachelor's degree (68.6%, n=371). More than half of the respondents were aged 18 to 29 years (58.8%, n=318) and were single (57.5%, n=311), and more than one-third of them were residing in the Western region (39.9%, n=216). The most commonly reported skin conditions included acne (26.8%, n=145) and eczema (12.8%, n=69, Table 1).

**Table 1:** Demographic characteristics and the history of skin conditions The data has been represented as N, %; p-value is considered significant (p<0.05)

Parameter	Category	N (%)
Gender	Male	153 (28.3%)
	Female	388 (71.7%)
Nationality	Saudi	504 (93.2%)
	Non-Saudi	37 (6.8%)
Age (year)	18-29	318 (58.8%)
	30-39	70 (12.9%)
	40-49	40 (7.4%)
	50-59	56 (10.4%)
	≥ 60	57 (10.5%)
Residential area	Northern region	85 (15.7%)
	Southern region	105 (19.4%)
	Eastern region	53 (9.8%)
	Western region	216 (39.9%)
	Central region	82 (15.2%)
Marital status	Single	311 (57.5%)
	Married	217 (40.1%)
	Other	13 (2.4%)
Educational level	Middle	6 (1.1%)
	Secondary	81 (15.0%)
	Diploma	38 (7.0%)
	Bachelor	371 (68.6%)
	Master/Doctorate	40 (7.4%)
	Other	5 (0.9%)
Occupation	Student	202 (37.3%)
	Employee	171 (31.6%)
	Unemployed	78 (14.4%)
	Retired	70 (12.9%)
	Other	20 (3.7%)
Monthly income	No income	119 (22.0%)
	< 3000	156 (28.8%)
	3000 - 7000	61 (11.3%)
	7001 - 12000	78 (14.4%)
	12001 - 18000	52 (9.6%)
	18001 - 25000	39 (7.2%)
	> 25000	36 (6.7%)
History of skin conditions	Psoriasis	23 (4.3%)
	Eczema	69 (12.8%)
	Acne	145 (26.8%)
	Baldness	32 (5.9%)
	Vitiligo	9 (1.7%)
	Rash (Urticaria)	15 (2.8%)
	Seborrheic dermatitis	4 (0.7%)

Description of stress

**Figure 1:**
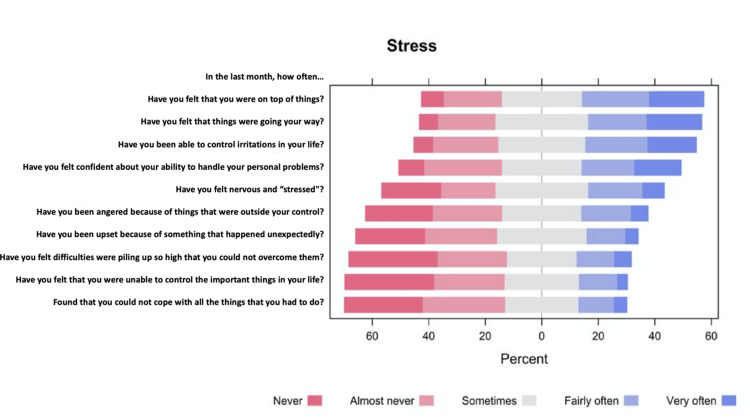
Participants' responses to the perceived stress scale The data has been represented as N and %; the p-value is considered significant (p<0.05)

**Figure 2:**
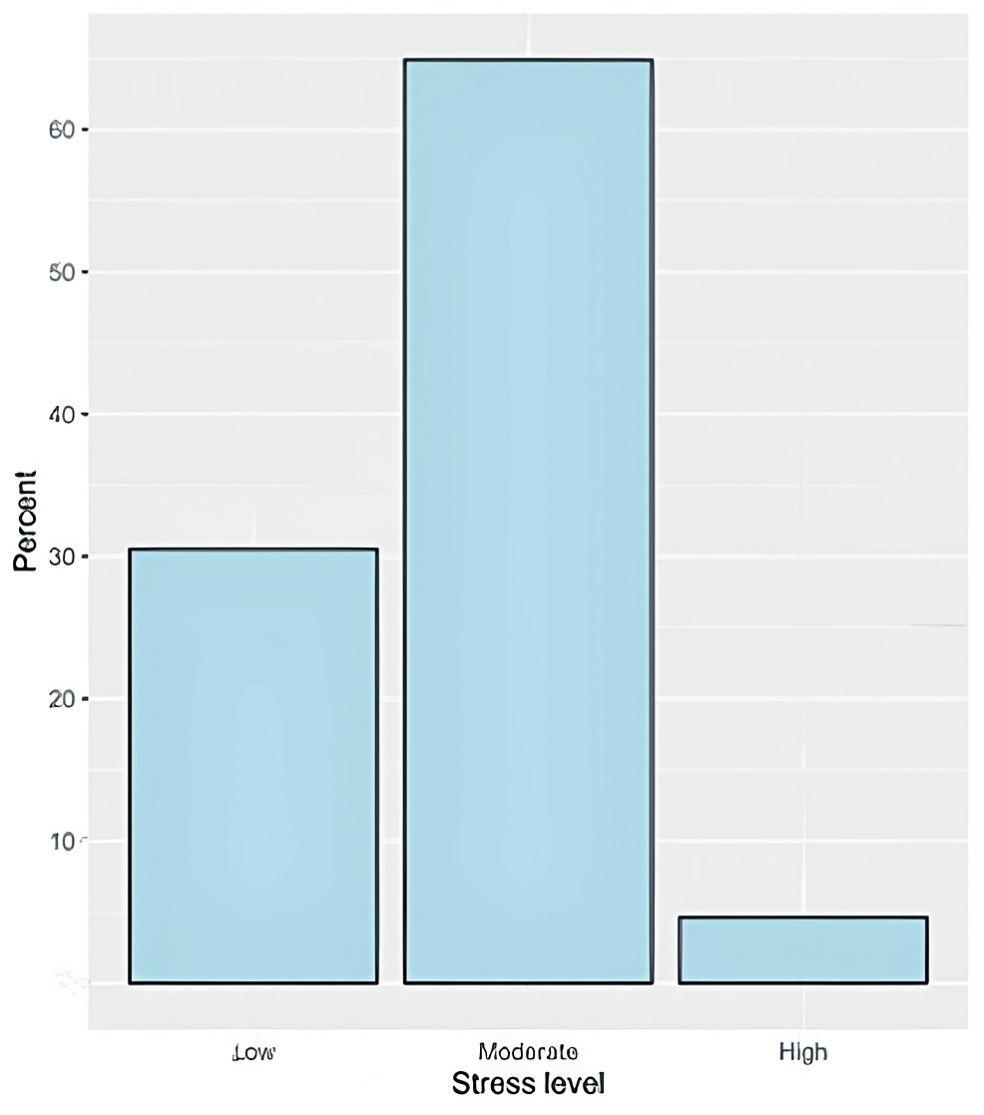
The percentages of stress levels among participants The data has been represented as N and %

In general, the stress scale showed very good internal consistency, as indicated by a Cronbach's alpha value of 0.891 (15 items) (Table 2). Participants' responses to the PSS are illustrated in Figure 1. Regarding the stress score, the median (IQR) score was 18 (12 to 21) with a minimum of 0 and a maximum of 40. Stress was low in (30.5%, n=165), moderate in (64.9%, n=351), and high in (4.6%, n=25) of the respondents (Figure 2). Results of the inferential analysis showed that stress levels differed significantly based on the residential region (p = 0.005), educational level (p = 0.045), and history of eczema (p = 0.012) and acne (p = 0.004, Table 3).

**Table 2:** Description of the scores of stress and self-reported skin symptoms The data has been represented as IQR; the p-value is considered significant (p<0.05) IQR: Interquartile range

Parameter	Items	Cronbach's alpha	Median (IQR)	Min – Max
Perceived Stress Scale	10	0.891	18.0 (12.0 to 21.0)	0.0 – 40.0
Self-Reported Skin Complaints Questionnaire	15	0.859	1.3 (1.1 to 1.7)	1.0 – 3.3

**Table 3:** Factors associated with stress levels among participants under study The data has been represented as N and %; the p-value is considered significant (p<0.05)

Parameter	Category	Stress level			
		Low, N = 165	Moderate, N = 351	High, N = 25	p-value
Gender	Male	48 (31.4%)	99 (64.7%)	6 (3.9%)	0.914
	Female	117(30.2%)	252(64.9%)	19(4.9%)	
Nationality	Saudi	157(31.2%)	322(63.9%)	25(5.0%)	0.188
	Non-Saudi	8 (21.6%)	29 (78.4%)	0 (0.0%)	
Age (year)	18-29	92 (28.9%)	208(65.4%)	18(5.7%)	0.665
	30-39	17 (24.3%)	50 (71.4%)	3 (4.3%)	
	40-49	15 (37.5%)	24 (60.0%)	1 (2.5%)	
	50-59	19 (33.9%)	36 (64.3%)	1 (1.8%)	
	≥ 60	22 (38.6%)	33 (57.9%)	2 (3.5%)	
Residential area	Northern region	20 (23.5%)	61 (71.8%)	4 (4.7%)	0.005
	Southern region	49 (46.7%)	51 (48.6%)	5 (4.8%)	
	Eastern region	21 (39.6%)	29 (54.7%)	3 (5.7%)	
	Western region	55 (25.5%)	153(70.8%)	8 (3.7%)	
	Central region	20 (24.4%)	57 (69.5%)	5 (6.1%)	
Marital status	Single	89 (28.6%)	207(66.6%)	15(4.8%)	0.660
	Married	72 (33.2%)	136(62.7%)	9 (4.1%)	
	Other	4 (30.8%)	8 (61.5%)	1 (7.7%)	
Educational level	Middle	5 (83.3%)	1 (16.7%)	0 (0.0%)	0.045
	Secondary	31 (38.3%)	48 (59.3%)	2 (2.5%)	
	Diploma	16 (42.1%)	21 (55.3%)	1 (2.6%)	
	Bachelor	103(27.8%)	248(66.8%)	20(5.4%)	
	Master/Doctorate	9 (22.5%)	30 (75.0%)	1 (2.5%)	
	Other	1 (20.0%)	3 (60.0%)	1(20.0%)	
Occupation	Student	66 (32.7%)	123(60.9%)	13(6.4%)	0.226
	Employee	39 (22.8%)	125(73.1%)	7 (4.1%)	
	Unemployed	29 (37.2%)	46 (59.0%)	3 (3.8%)	
	Retired	24 (34.3%)	44 (62.9%)	2 (2.9%)	
	Other	7 (35.0%)	13 (65.0%)	0 (0.0%)	
Monthly income	No income	41 (34.5%)	75 (63.0%)	3 (2.5%)	0.163
	< 3000	50 (32.1%)	94 (60.3%)	12(7.7%)	
	3000 - 7000	21 (34.4%)	38 (62.3%)	2 (3.3%)	
	7001 - 12000	16 (20.5%)	60 (76.9%)	2 (2.6%)	
	12001 - 18000	20 (38.5%)	30 (57.7%)	2 (3.8%)	
	18001 - 25000	7 (17.9%)	29 (74.4%)	3 (7.7%)	
	> 25000	10 (27.8%)	25 (69.4%)	1 (2.8%)	
History of skin conditions	Psoriasis	3 (13.0%)	19 (82.6%)	1 (4.3%)	0.139
	Eczema	11 (15.9%)	54 (78.3%)	4 (5.8%)	0.012
	Acne	29 (20.0%)	106(73.1%)	10(6.9%)	0.004
	Baldness	6 (18.8%)	24 (75.0%)	2 (6.2%)	0.251
	Vitiligo	3 (33.3%)	5 (55.6%)	1(11.1%)	0.376
	Rash (Urticaria)	6 (40.0%)	9 (60.0%)	0 (0.0%)	0.787
	Seborrheic dermatitis	3 (75.0%)	1 (25.0%)	0 (0.0%)	0.244
	Other	19 (43.2%)	22 (50.0%)	3 (6.8%)	0.065

Description of self-reported skin symptoms and the associated factors

**Figure 3:**
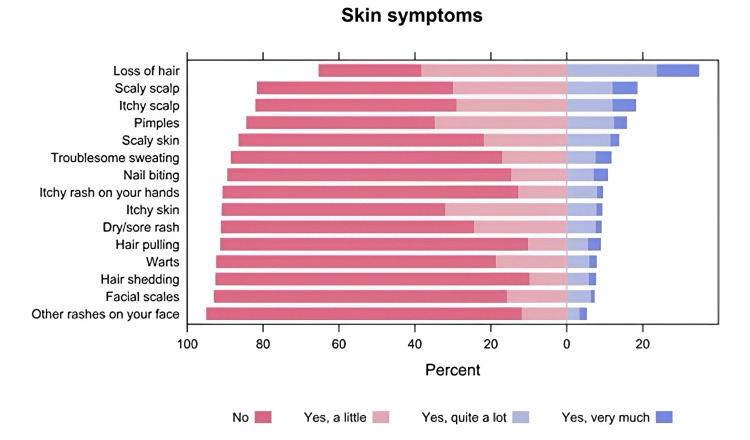
Participants' responses to the self-reported skin symptom scale The data has been represented as N and %; the p-value is considered significant (p<0.05)

Reliability analysis showed that the Cronbach's alpha value of the self-reported skin symptoms scale was 0.859 (Table 2). Participants answered "yes, quite a lot" or "yes, very much" more frequently for loss of hair (34.7%, n=187), scaly scalp (18.5%, n=100), and itchy scalp (18.1%, n=97, Figure 3).

The median (IQR) score of skin symptoms among the overall cohort was 1.3 (1.1 to 1.7), and the minimum and maximum scores were 1 and 3.3, respectively (Table 2). Average scores of skin symptoms differed significantly based on participants' ages (p < 0.001), residential regions (p < 0.001), marital status (p < 0.001), educational level (p < 0.001), occupation (p < 0.001), and monthly income (p < 0.001), as well as having the following skin conditions: psoriasis (p < 0.001), eczema (p < 0.001), acne (p < 0.001), rash (p < 0.001), and baldness (p = 0.041). Furthermore, average skin symptom scores were significantly higher among participants with a high stress level (median = 1.6, IQR = 1.4, 1.8) compared to those with moderate stress (median = 1.4, IQR = 1.2, 1.8) and low stress (median = 1.4, IQR = 1.1, 1.4, p < 0.001, Table 4).

**Table 4:** Demographic-based differences in the average skin symptom scores The data has been represented as IQR; the p value is considered significant (p<0.05, p<0.001)

Parameter	Category	Median (IQR)	p-value
Gender	Male	1.3 (1.1, 1.7)	0.154
	Female	1.4 (1.2, 1.7)	
Nationality	Saudi	1.3 (1.1, 1.7)	0.955
	Non-Saudi	1.3 (1.2, 1.7)	
Age (year)	18-29	1.5 (1.3, 1.8)	<0.001
	30-39	1.3 (1.1, 1.6)	
	40-49	1.2 (1.1, 1.4)	
	50-59	1.3 (1.1, 1.6)	
	≥ 60	1.1 (1.1, 1.3)	
Residential area	Northern area	1.9 (1.3, 2.6)	<0.001
	Southern area	1.4 (1.2, 1.7)	
	Eastern area	1.3 (1.1, 1.7)	
	Western area	1.3 (1.1, 1.5)	
	Middle area	1.4 (1.2, 1.6)	
Marital status	Single	1.4 (1.2, 1.8)	<0.001
	Married	1.3 (1.1, 1.5)	
	Other	1.4 (1.2, 1.6)	
Educational level	Middle	1.1 (1.1, 1.5)	<0.001
	Secondary	1.3 (1.1, 1.5)	
	Diploma	1.5 (1.2, 1.6)	
	Bachelor	1.4 (1.2, 1.8)	
	Master/Doctorate	1.2 (1.1, 1.4)	
	Other	1.7 (1.2, 2.1)	
Occupation	Student	1.4 (1.2, 1.7)	<0.001
	Employee	1.5 (1.2, 2.2)	
	Unemployed	1.3 (1.1, 1.6)	
	Retired	1.2 (1.1, 1.3)	
	Other	1.4 (1.2, 1.6)	
Monthly income	No income	1.3 (1.1, 1.6)	<0.001
	< 3000	1.3 (1.2, 1.7)	
	3000 - 7000	1.4 (1.1, 1.6)	
	7001 - 12000	1.9 (1.3, 2.5)	
	12001 - 18000	1.3 (1.2, 1.7)	
	18001 - 25000	1.3 (1.1, 1.5)	
	> 25000	1.2 (1.1, 1.4)	
Psoriasis	No	1.3 (1.1, 1.7)	<0.001
	Yes	1.9 (1.6, 2.6)	
Eczema	No	1.3 (1.1, 1.6)	<0.001
	Yes	1.7 (1.4, 2.1)	
Acne	No	1.3 (1.1, 1.5)	<0.001
	Yes	1.7 (1.3, 2.3)	
Baldness	No	1.3 (1.1, 1.7)	0.041
	Yes	1.5 (1.2, 2.2)	
Vitiligo	No	1.3 (1.1, 1.7)	0.118
	Yes	1.5 (1.3, 1.9)	
Rash (Urticaria)	No	1.3 (1.1, 1.7)	<0.001
	Yes	2.3 (1.8, 2.6)	
Seborrheic dermatitis	No	1.3 (1.1, 1.7)	0.392
	Yes	1.4 (1.4, 1.5)	
Other	No	1.3 (1.1, 1.7)	0.641
	Yes	1.4 (1.2, 1.7)	
Stress level	Low	1.3 (1.1, 1.4)	<0.001
	Moderate	1.4 (1.2, 1.8)	
	High	1.6 (1.4, 1.8)	

Predictors of high scores of skin symptoms

Based on the multivariable regression analysis, average skin symptoms were independently lower among participants aged 30 to 39 years (beta = -0.12, 95%CI, -0.24 to -0.01, p = 0.044), 40 to 49 years (beta = -0.24, 95%CI, -0.38 to -0.10, p < 0.001), and ≥ 60 years (beta = -0.23, 95%CI, -0.43 to -0.03, p = 0.022) and those who were married (beta = -0.10, 95%CI, -0.20 to -0.01, p = 0.039). Additionally, residents of the following regions had independently lower scores of skin symptoms: Southern (beta = -0.22, 95%CI, -0.33 to -0.11, p < 0.001), Eastern (beta = -0.24, 95%CI, -0.38 to -0.11, p < 0.001), Western (beta = -0.29, 95%CI, -0.40 to -0.19, p < 0.001), and Central regions (beta = -0.23, 95%CI, -0.35 to -0.12, p < 0.001). Conversely, higher skin symptoms scores were independently predicted by being unemployed (beta = 0.19, 95%CI, 0.08 to 0.30, p = 0.001), having monthly incomes of 7001 - 12000 (beta = 0.28, 95%CI, 0.14 to 0.43, p < 0.001) and 12001 - 18000 (beta = 0.20, 95%CI, 0.05 to 0.35, p = 0.008) and being diagnosed with the following skin conditions: psoriasis (beta = 0.39, 95%CI, 0.24 to 0.54, p < 0.001), eczema (beta = 0.23, 95%CI, 0.14 to 0.32, p < 0.001), acne (beta = 0.22, 95%CI, 0.15 to 0.29, p < 0.001), baldness (beta = 0.15, 95%CI, 0.02 to 0.28, p = 0.020), and rash (beta = 0.39, 95%CI, 0.21 to 0.57, p < 0.001). Of note, having higher stress levels was a significant predictor of a high skin symptoms score, including moderate stress (beta = 0.15, 95%CI, 0.09 to 0.22, p < 0.001) and high stress (beta = 0.27, 95%CI, 0.12 to 0.42, p < 0.001, Table 5).

**Table 5:** Predictors of the high skin symptom score The data has been represented as beta and CI; the p-value is considered significant (p<0.05, p<0.001)

Parameter	Category	Beta	95% CI	p-value
Age (year)	18-29	—	—	
	30-39	-0.12	-0.24, 0.00	0.044
	40-49	-0.24	-0.38, -0.10	<0.001
	50-59	-0.11	-0.25, 0.04	0.145
	≥ 60	-0.23	-0.43, -0.03	0.022
Residential region	Northern region	—	—	
	Southern region	-0.22	-0.33, -0.11	<0.001
	Eastern region	-0.24	-0.38, -0.11	<0.001
	Western region	-0.29	-0.40, -0.19	<0.001
	Central region	-0.23	-0.35, -0.12	<0.001
Marital status	Single	—	—	
	Married	-0.10	-0.20, -0.01	0.039
	Other	-0.03	-0.25, 0.18	0.760
Educational level	Middle	—	—	
	Secondary	-0.23	-0.53, 0.06	0.121
	Diploma	-0.09	-0.39, 0.21	0.563
	Bachelor	-0.21	-0.50, 0.08	0.153
	Master/Doctorate	-0.35	-0.66, -0.03	0.030
	Other	0.11	-0.32, 0.53	0.620
Occupation	Student	—	—	
	Employee	0.13	0.01, 0.25	0.041
	Unemployed	0.19	0.08, 0.30	0.001
	Retired	0.09	-0.10, 0.29	0.349
	Other	0.15	-0.04, 0.35	0.116
Monthly income	No income	—	—	
	< 3000	0.00	-0.09, 0.09	0.986
	3000 - 7000	0.05	-0.10, 0.19	0.522
	7001 - 12000	0.28	0.14, 0.43	<0.001
	12001 - 18000	0.20	0.05, 0.35	0.008
	18001 - 25000	0.08	-0.09, 0.24	0.352
	> 25000	0.08	-0.10, 0.25	0.387
Psoriasis	No	—	—	
	Yes	0.39	0.24, 0.54	<0.001
Eczema	No	—	—	
	Yes	0.23	0.14, 0.32	<0.001
Acne	No	—	—	
	Yes	0.22	0.15, 0.29	<0.001
Baldness	No	—	—	
	Yes	0.15	0.02, 0.28	0.020
Rash	No	—	—	
	Yes	0.39	0.21, 0.57	<0.001
Stress level	Low	—	—	
	Moderate	0.15	0.09, 0.22	<0.001
	High	0.27	0.12, 0.42	<0.001

## Discussion

The current study provides evidence of the strong relationship that exists between high stress levels and common skin disorders. The prevalence of psoriasis, eczema, acne, baldness, vitiligo, and rash was higher among individuals with moderate and high stress levels. Furthermore, the average skin symptom scores were significantly higher among participants with high stress levels than among those with moderate and low stress. Therefore, a variety of common skin disorders may manifest when individuals experience PS. We found that acne and eczema were the most commonly reported skin conditions that were statically and significantly associated with a moderate stress level (p = 0.004 and p = 0.012, respectively). Our findings are in line with the results of other studies on this topic [27-32].

In this study, acne was one of the most common skin conditions related to stress; however, other studies demonstrated that increased stress was not associated with acne emergence but with acne severity [28,32]. Even though stress and anxiety play a major role in causing acne, the study showed that the severity of acne was correlated with the stress level. Moreover, we also found that eczema was significantly associated with the level of stress [29].

The perceived stress levels were higher among women, consistent with the results of earlier studies showing that stress during daily activities and at school had a particular impact on females [23,33,34]. In this study, we found that diploma, bachelor, and master/doctorate-level education was associated with the highest perceived stress levels. In contrast, another study found no significant relationship between academic standing and the student's PS levels [23]. One study reported that heightened stress in undergraduate students was associated with pruritus, alopecia, oily/waxy/flaky patches on the scalp, hyperhidrosis, scaly skin, onychophagia, trichotillomania, and itchy rash on hands [35].

Psychological and psychiatric issues are associated with more than 30% of all dermatologic illnesses [36]. Numerous studies have shown that various skin conditions are caused and exacerbated by chronic PS, associated with psychiatric disorders or certain personality traits [13,14,37]. The role of PS has been reported in a variety of skin conditions, including atopic dermatitis, alopecia areata, seborrheic dermatitis, urticaria, psoriasis, telogen effluvium, acne vulgaris, pruritus, prurigo nodularis, and lichen planus [13,18,37,38].

The connection between PS and skin problems is mediated by a complicated neuro-immuno-cutaneous-endocrine network. The HPA axis and the autonomic nervous system (sympathetic and cholinergic) are the two main neuroendocrine systems that are activated in response to PS exposure. The HPA axis hormones are altered by PS, along with the release of stress-related mediators such as neuropeptides and cytokine profiles. PS consequently affects the immunological response [6]. The immune system's cutaneous and skin-infiltrating cells have receptors for stress mediators. Additionally, the skin is controlled by a peripheral HPA axis that is equivalent to the central HPA axis [6,39]. As a result, when the skin is subjected to stress, it produces glucocorticoids, corticotropin-releasing hormone, and adrenocorticotropic hormone locally, resulting in an increase in the number of substance P-positive nerve fibers [40]. Skin disorders caused by chronic stress are largely attributed to neuroendocrine and immunological changes that limit the skin's capacity to respond to environmental challenges [39]. Moreover, chronic stress can lead to autoimmune disorders. Mast cells play a crucial in the immunological reactions to stress by inducing neurogenic inflammation; these stress-induced changes in the skin may play a role in the exacerbation of skin disease [39].

The main strength of our study is the sample size of 541 participants. One of the main limitations of this study is that the causal relationship between the variables could not be demonstrated due to the cross-sectional study design. The use of the SSCQ rather than a dermatologist diagnosis is another limitation of our study. However, the SSCQ is a validated instrument.

## Conclusions

The general population of Saudi Arabia reported multiple skin symptoms associated with stress. PS can cause various common skin conditions including loss of hair, eczema, and acne. This study highlights the importance of assessing common skin problems in the general population in the KSA and their strong association with PS. Various skin conditions including loss of hair, eczema, and acne can be caused by PS. Dermatologists should be aware of the context of PS when assessing patients with these conditions.
